# The migration of paraxial and lateral plate mesoderm cells emerging from the late primitive streak is controlled by different Wnt signals

**DOI:** 10.1186/1471-213X-8-63

**Published:** 2008-06-09

**Authors:** Dylan Sweetman, Laura Wagstaff, Oliver Cooper, Cornelis Weijer, Andrea Münsterberg

**Affiliations:** 1 School of Biological Sciences, University of East Anglia, Norwich Research Park, Norwich, NR4 7TJ, UK; 2 Biomedical Research Centre, University of East Anglia, Norwich Research Park, Norwich, NR4 7TJ, UK; 3 Centre for Neuroregeneration Research, Harvard Medical School, McLean Hospital, Belmont, MA 02478, USA; 4 College of Life Sciences Biocentre, MSI/WTB complex, University of Dundee, Dow Street, Dundee, DD1 5EH, UK

## Abstract

**Background:**

Co-ordinated cell movement is a fundamental feature of developing embryos. Massive cell movements occur during vertebrate gastrulation and during the subsequent extension of the embryonic body axis. These are controlled by cell-cell signalling and a number of pathways have been implicated. Here we use long-term video microscopy in chicken embryos to visualize the migration routes and movement behaviour of mesoderm progenitor cells as they emerge from the primitive streak (PS) between HH stages 7 and 10.

**Results:**

We observed distinct cell movement behaviours along the length of the streak and determined that this is position dependent with cells responding to environmental cues. The behaviour of cells was altered by exposing embryos or primitive streak explants to cell pellets expressing Wnt3a and Wnt5a, without affecting cell fates, thus implicating these ligands in the regulation of cell movement behaviour. Interestingly younger embryos were not responsive, suggesting that Wnt3a and Wnt5a are specifically involved in the generation of posterior mesoderm, consistent with existing mouse and zebrafish mutants. To investigate which downstream components are involved mutant forms of dishevelled (dsh) and prickle1 (pk1) were electroporated into the primitive streak. These had differential effects on the behaviour of mesoderm progenitors emerging from anterior or posterior regions of the streak, suggesting that multiple Wnt pathways are involved in controlling cell migration during extension of the body axis in amniote embryos.

**Conclusion:**

We suggest that the distinct behaviours of paraxial and lateral mesoderm precursors are regulated by the opposing actions of Wnt5a and Wnt3a as they leave the primitive streak in neurula stage embryos. Our data suggests that Wnt5a acts via prickle to cause migration of cells from the posterior streak. In the anterior streak, this is antagonised by Wnt3a to generate non-migratory medial mesoderm.

## Background

The production and migration of mesoderm from the primitive streak is a central event in amniote embryos. Extensive fate mapping has been done focussing in the chicken embryo on early to mid-gastrula (HH3 to HH4) as well as late gastrula/neurula stages (HH4 to HH7) [1-4]. These experiments showed that paraxial and lateral plate mesoderm is first generated during mid-gastrula stages and revealed that the anterior-posterior position of cells in the streak correlates with their eventual medio-lateral position within the mesoderm. However, the behaviour of cells as they leave the primitive streak was not visualized directly and their movement tracks were inferred from cell labelling and end point analyses. To address this, we previously established long-term video microscopy of individual GFP labelled cells in whole chick embryos cultured in modified New culture (EC culture) [5-8]. Using this approach, we demonstrated that at HH4 Fgf mediated signals control the movement of mesoderm progenitor cells. In particular, cell movement is directed by Fgf-8 mediated repulsion from the primitive streak and attraction towards the midline in response to Fgf-4 from the notochord. [6] These findings were consistent with the reported expression patterns for Fgf-4 and Fgf-8 in the primitive streak and with the phenotype of mice mutant in Fgf8 or Fgfr1 where cells remain 'trapped' in the streak [9-11].

A similar phenotype was observed in mouse embryos mutant for the frizzled co-receptors, Lrp5 and Lrp6, suggesting a requirement for Wnt signaling in gastrulation. Furthermore, this work provided genetic evidence in support of a molecular link between Fgf and Wnt signalling pathways in patterning nascent mesoderm [12]. Interestingly, mice mutant for Wnt3a gastrulate normally; however the generation of somites from the late primitive streak is affected in these mice. This suggested that the first non-redundant requirement for Wnt3a is during caudal somite and tail bud formation [13] and implied the presence of additional signals, such as FGFs, and potentially other Wnt ligands such as Wnt8c, able to compensate for Wnt3a during early but not later stages.

Screens in zebrafish embryos identified a number of mutants that affect gastrulation [14] and further experiments in non-amniote embryos demonstrated a role for non-canonical signalling. In particular, signalling through dishevelled and the planar cell polarity (PCP) pathway controls convergent extension (CE) type movements [15]. Mis-expression of either full-length prickle1 (pk1) or pk1-deletion-mutants disrupt convergent extension in *Xenopus *and zebrafish gastrulation [16] suggesting that pk1 is involved in PCP signalling [15]. In both zebrafish and *Xenopus *silberblick/Wnt11 function is required in early gastrulation for correct cell movements and extension of axial tissues, which is mediated through medio-lateral cell intercalation [17,18]. In this context, Wnt11 acts through dishevelled and controls the Rab5c mediated endocytosis of E-cadherin thereby regulating cell cohesion [19]. Furthermore, genetic analysis has implicated non-canonical signalling mediated by pipetail/Wnt5 in posterior body development in zebrafish [20]. The latter is consistent with findings in mice, where a loss-of-function mutation of Wnt5a leads to the progressive reduction of caudal structures, which results in the inability to extend the anterior-posterior axis [21]. Notably in both Wnt3a and Wnt5a mutants, early gastrulation movements occur normally and defects are only observed later during axis extension [13,21].

Here we used GFP labelling and live imaging to investigate the movement behaviour and trajectories of mesoderm progenitor cells emerging from the late primitive streak in amniote embryos, where much less is known about the control of cell behaviour. The chick embryo develops as a flat disc, is easily accessible and can be cultured on a semi-solid agarose-albumin mixture, which makes it suitable for manipulations and time-lapse imaging. Movies recorded of whole embryos and primitive streak explants showed that prospective paraxial and lateral plate mesoderm cells, emerging from the anterior and posterior streak respectively, exhibit different movement characteristics. We show that these are governed by extrinsic cues and find that Wnt3a and Wnt5a have distinct and opposing effects on the movement behaviour of these cells at HH stage 7 or older but not in younger embryos. Functional interference experiments suggest a dishevelled-dependent pathway is required for efficient movement and extension of paraxial mesoderm cells after they emerge from the anterior streak. In contrast, a mutant dishevelled protein did not inhibit the movement behaviour of cells that originated from the posterior streak. Furthermore, we examined the role of pk1. Because pk1 has been implicated in PCP signalling [15] it was unexpected to find that mis-expression of pk1 or pk1-deletion-mutants did not affect the convergent extension movements of cells derived from anterior streak, however, they efficiently blocked the migration of cells originating from the posterior streak. Together these studies illustrate that multiple Wnt pathways are involved in the control of cell movement behaviours during late primitive streak stages in amniote embryos.

## Results

### Movement trajectories of mesoderm progenitors emerging from the late primitive streak

**Figure 1:**
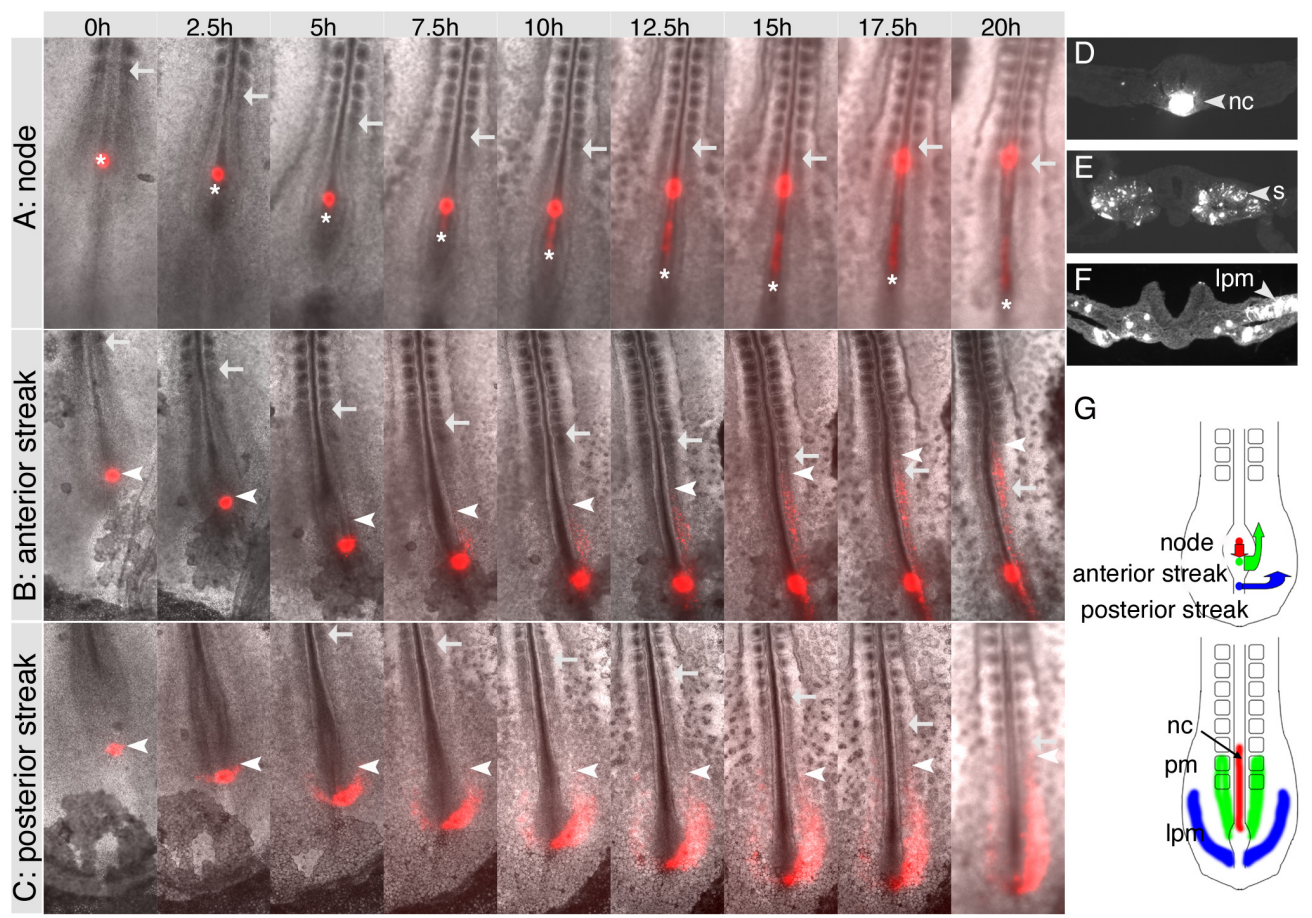
**DiI labelling in HH8 embryos reveals behaviour and trajectories of cells from the primitive streak**. (A, B, C) Long-term time-lapse imaging of embryos labelled in Hensen's node (A), the anterior (B) or posterior primitive streak (C) with DiI. Embryos were imaged for 20 hours and still pictures are shown at intervals of 2.5 hours. (D, E, F) Transverse sections of embryos labelled in Hensen's node (D), anterior (E) or posterior streak (F). (G) Summary of cell fates. Red represents Hensen's node and notochord (nc), green represents anterior primitive streak cells and paraxial mesoderm (pm) and blue represents posterior primitive streak cells and lateral plate mesoderm (lpm). n, node; nc, notochord; pm, paraxial mesoderm; s, somites. In (A) asterisks indicate the position of the node which regresses during axis extension. In (A, B, C) arrows show the most recently formed somite and arrowheads in (B, C) indicate the position of the most anterior DiI-labelled cells.

To determine the movement trajectories of mesoderm cells emerging from the late primitive streak we performed injections with the vital dye, DiI, into HH stage 8 to 12 chick embryos in EC culture. Cell movement was then monitored over 20 hours by time-lapse video microscopy on a Zeiss Axiovert inverted microscope. Consistent with established fate maps we found that cells from the node remained in the midline and contributed to the notochord as the node regressed (Fig. 1A, D). Cells residing behind the node, in the anterior part of the primitive streak, contributed to paraxial mesoderm after leaving the streak (Fig. 1B, E) and cells from the central and posterior regions of the streak produced intermediate and lateral plate mesoderm (Fig. 1C, F; Supplemental Movie 1) [1,2,4,22]. Movement tracks are schematically represented (Fig. 1G). Observation of cell movements revealed striking differences in the behaviour of different mesoderm progenitors. Prospective paraxial mesoderm cells from the anterior streak showed little or no lateral movement after leaving the streak and appear to be displaced by the regressing node as the axis extends. In contrast, intermediate and lateral mesoderm progenitors, located more posteriorly within the streak, migrated actively in a lateral direction before converging towards the midline and participating in axis extension. The extent of lateral movement was more pronounced in progressively more posterior streak cells. These observations are consistent with our previous findings and with a recent quantitative analysis of movement behaviour at earlier stages (HH4, HH5) [6,23].

### Wnt-3a affects cell movement behaviour in vivo

**Figure 2:**
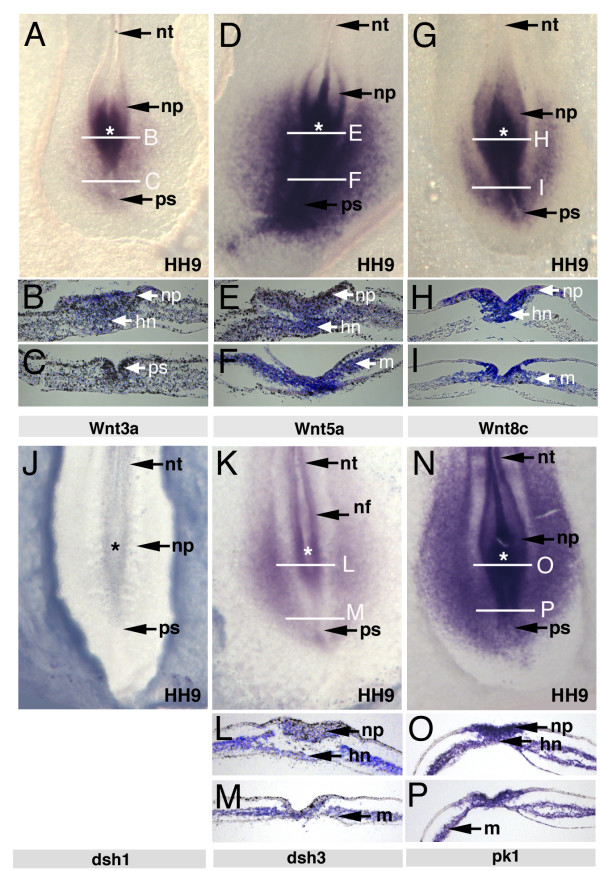
**Differential expression of Wnt ligands and Wnt pathway components in the late primitive streak**. (A) *Wnt3a in situ *hybridisation, white lines indicate the levels of section in (B) through the anterior streak with staining in the neural plate (C), section through the posterior streak with low levels of *Wnt3a *transcripts. (D) *Wnt5a in situ *hybridisation, white lines indicate the levels of section in (E) through the anterior and (F) through the posterior streak indicating widespread expression of *Wnt5a *in neural plate and mesoderm. (G) *Wnt8c in situ *hybridisation, white lines indicate the levels of section in (H) through the anterior and (I) through the posterior streak indicating expression of *Wnt8c *in neural plate and mesoderm. (J) Dishevelled-1 was not expressed in posterior regions of HH9 embryos but (K) dishevelled-3 and (N) prickle-1 showed similar expression in neural folds, lateral plate mesoderm and low levels in the primitive streak White lines indicate the levels of section in L, O (anterior), M and P (posterior) streak of dishevelled-3 and prickle-1. HH stage is indicated on each panel. hn, Hensen's node; m, mesoderm; nf, neural fold; np, neural plate; nt, neural tube; ps, primitive streak; * asterisks indicate position of the node.

A series of heterotopic and heterochronic grafts showed that cells adjust to their new environment and confirmed that cell movement behaviour is determined by extrinsic cues (not shown). To investigate the potential role of Wnt signalling in controlling movement behaviour we examined the expression patterns of several Wnt ligands and non-canonical signalling components in posterior regions of HH7-12 embryos. Expression of Wnt3a, Wnt5a and Wnt8c transcripts was detected in posterior regions of chick embryos (Fig. 2). Whole mount embryos and transverse sections showed that Wnt3a was expressed in the neural plate overlying the anterior streak with very low levels of transcripts detected in the posterior primitive streak (Fig. 2A–C). Wnt5a and Wnt8c were both expressed throughout the neural plate and primitive streak. Wnt5a transcripts were present throughout the entire mesoderm posteriorly (Fig. 2D–F) while Wnt8c was expressed in migrating lateral plate mesoderm progenitors but not in paraxial mesoderm progenitors (Fig. 2G–I). In addition, we examined the expression of some downstream components of Wnt signalling. Dishevelled-1 transcripts were not detected in the primitive streak (Fig. 2J) but dishevelled-3 was expressed both in the streak and in the adjacent mesoderm (Fig. 2K, L, M). Pk1 was also expressed in the neural plate, the primitive streak and the adjacent mesoderm (Fig. 2N, O, P and [24]).

**Figure 3:**
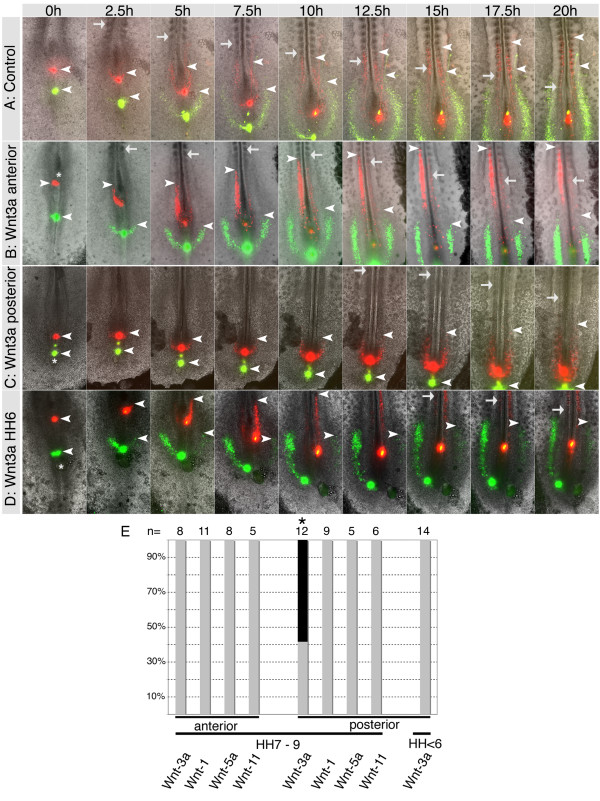
**Ectopic *Wnt3a *alters the movement behaviour of posterior primitive streak cells *in vivo***. (A) Injection of DiI (red) and DiO (green) into the anterior and posterior primitive streak followed by long-term time-lapse video microscopy shows the normal cell movement patterns of paraxial and lateral plate mesoderm progenitors. (B) A pellet of cells expressing Wnt3a adjacent to the anterior primitive streak had no effect on cell movement behaviour. (C) Implanting Wnt3a cells into the posterior streak blocked cell migration and production of lateral plate mesoderm in HH7-9 embryos. (D) Wnt3a cells implanted into the posterior streak at HH6 did not affect cell migration. (E) Graphical illustration of the effects of Wnt cell pellets on primitive streak cell behavior; grey shading indicates normal behavior, black shading indicates altered behavior. Only posterior grafts of Wnt3a altered normal movements in HH7-9 embryos. Asterisks indicate the position of the cell pellet at 0 h (B, C, D). White arrows indicate the most recently formed somite and arrowheads indicate the most anterior DiI and DiO labelled cells that have left the primitive streak.

The expression patterns of Wnts suggested that they could function to control the behaviour of primitive streak cells. In particular, the differential expression of Wnt3a was striking with high levels in the anterior and much lower levels in the posterior primitive streak. Thus, we determined whether exogenous Wnt3a could affect cell movement behaviour in the embryo. Embryos were labelled with DiI in the anterior and DiO in the posterior streak. In some cases cells were only observed to migrate from one side of the streak. This was due to asymmetric labelling and does not represent asymmetric migration of cells from the streak. The movement patterns of mesoderm progenitors in these embryos was identical to that observed previously in single labelled embryos (compare Fig. 3A with Fig. 1A–C) (see Additional file 1 for the original data used to generate this image sequence). Next, we grafted cell pellets expressing Wnt3a into the anterior primitive streak (Fig. 3B). Time-lapse video microscopy demonstrated that this did not affect the movement patterns or behaviour of cells emerging from the anterior primitive streak; cells travelled a similar distance from the DiI injection site at any given time point and paraxial and lateral mesoderm was generated normally (n = 8/8, Fig. 3B). However, when Wnt3a cells were grafted into the posterior streak of HH8-10 embryos the movement behaviour of lateral plate mesoderm progenitors was significantly altered and their lateral migration was almost completely blocked (n = 7/12, Fig. 3C). In these embryos, paraxial mesoderm progenitors were still generated from the anterior streak although axis extension was impaired, possibly due to the fact that efficient generation of lateral plate mesoderm from the posterior streak was inhibited. Wnt3a cells only affected cell migration in embryos at stages older than HH7. When Wnt3a cell pellets were grafted into the posterior primitive streak of younger embryos (between HH4 and 6) no effects on cell migration were observed (n = 14, Fig. 3D).

Implanting cells expressing either Wnt5a (n = 13), Wnt1 (n = 20) or Wnt11 (n = 11) into either the anterior or posterior primitive streak had no discernable effect on the movement of mesoderm progenitor cells (Fig. 3E). Expression of Wnt proteins in RatB1A fibroblasts was verified by Western blot using anti-HA antibodies. In all cases bands of the correct sizes were detected (data not shown, [25]).

**Figure 4:**
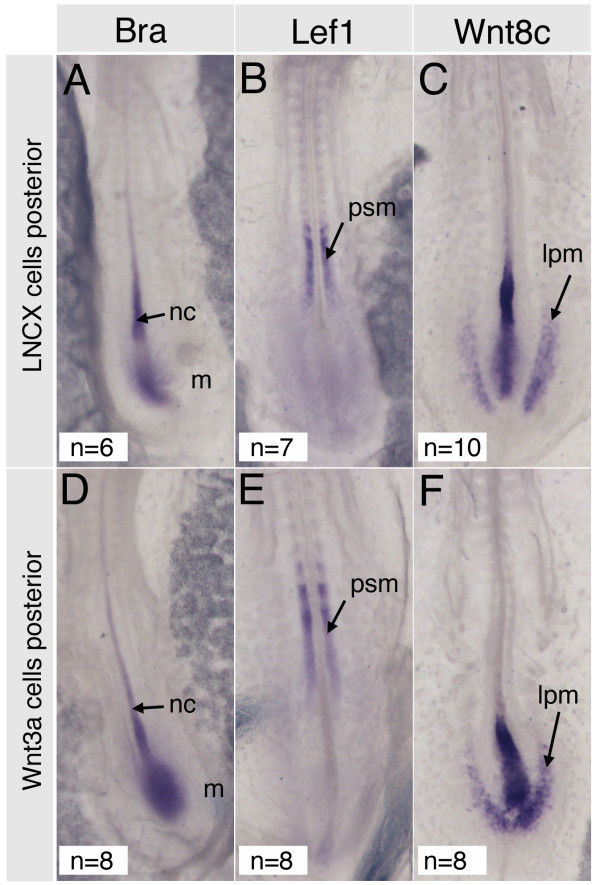
**Posterior grafts of Wnt3a cell pellets do not affect cell fate**. (A-C) Expression patterns after grafting Rat-B1a-LNCX control cells. (D-F) Expression patterns after grafting Rat-B1a-Wnt3a cells. (A, D) Brachyury, (B, E) Lef1, (C, F) Wnt8c. nc, notochord; m, mesoderm; psm, presegmented mesoderm; lpm, lateral plate mesoderm.

To exclude the possibility that altered cell movement behaviour resulted from changes in cell fate we examined expression of various mesoderm markers. We used probes for Brachyury, a marker for early mesoderm and notochord, Lef-1, a marker of paraxial mesoderm and Wnt8c, which is expressed in lateral mesoderm. Implantation of Wnt3a cell pellets or Rat-B1a-LNCX control pellets did not alter the expression of these genes (Fig 4, compare panels A-C with D-F). This suggested that effects on movement of posterior streak cells was unlikely to be secondary to changes in cell fate.

### Anterior and posterior primitive streak cells display different behaviour in explant cultures

**Figure 5:**
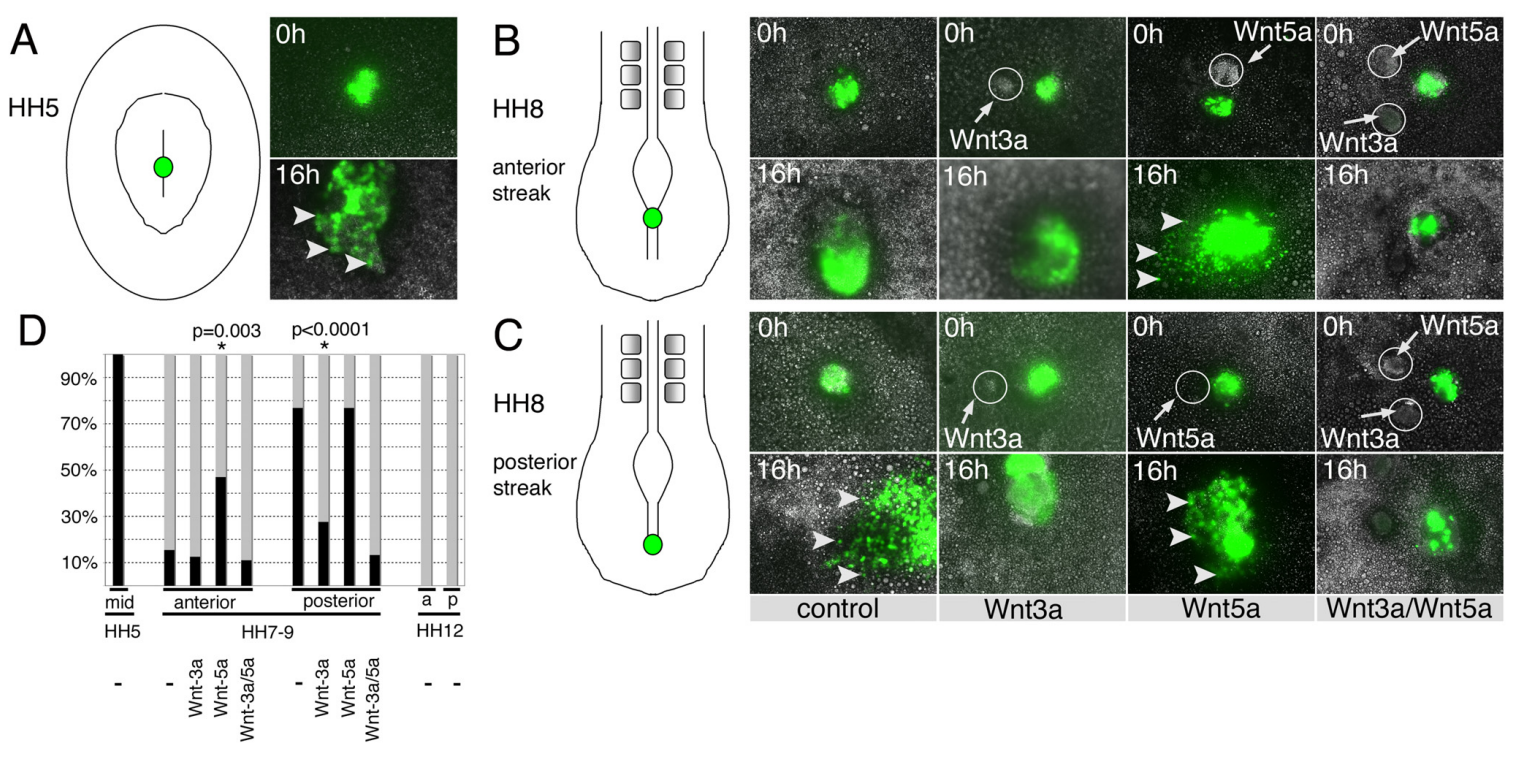
**The behaviour of anterior and posterior primitive streak explants is affected by Wnt3a and Wnt5a**. Embryos were electroporated with pCS2^+^GFP and following GFP expression explants were dissected from anterior and posterior primitive streak as indicated in the drawings. Explants were placed on the area opaca of a host embryo (HH4). Labelled cells were imaged overnight and still images are shown at zero and 16 hours. Timelapse sequences were scored as migratory if they contained individual cells migrating away from the explant. χ^2 ^analysis was used to determine if changes in explant behaviour were statistically significant. (A) Control explants from a HH5 embryo; cells are migratory. (B) Explants from the anterior primitive streak of a HH8 embryo; cells remained cohesive and few individual cells moved away from the explants. This behavior was not affected by Wnt3a expressing cells, but in response to Wnt5a the explants dispersed and more migrating cells were observed. Anterior explants exposed to both Wnt3a and Wnt5a remained cohesive. (C) Explants from the posterior primitive streak of a HH8 embryo contained many cells migrating away and most of the explant dispersed by the end of imaging. In the presence of Wnt3a expressing cells posterior explants remained cohesive. Wnt5a expressing cells had no effect and migrating cells were observed. Posterior explants exposed to both Wnt3a and Wnt5a remained cohesive. Arrowheads indicate cells that have migrated from explants, circles indicate positions of cell pellets. (D) Summary of explant data. χ^2 ^analyses showed that the altered behavior in response to Wnt5a or Wnt3a was statistically significant. Black bars indicate the percentage of explants showing migratory behaviour, grey bars indicate explants with non-migratory behaviour.

To examine the behaviour of paraxial and lateral plate mesoderm progenitor cells in a less complex environment, primitive streak cells were electroporated with an expression plasmid driving the expression of green fluorescent protein (GFP) from the viral CMV promoter (pCS2^+^GFP). Following GFP expression small grafts were excised and transferred onto the area opaca of unlabelled HH4 host embryos (as described in [6]) and monitored by time-lapse for 16 hours. Primitive streak cell explants from mid-gastrula embryos (HH4-5) had previously been examined in this ex-vivo system and were used as controls [6]. Time-lapse movies showed that HH5 mid-primitive streak cells actively migrated away from the explants, which were almost completely dispersed by the end of the culture period (n = 6, Fig. 5A, D). Explants derived from HH7-9 primitive streak showed different behaviours, which correlated with their anterior-posterior origin from within the streak. Anterior primitive streak explants remained cohesive and no individual cells separating from the explants were observed. However, the explants significantly expanded suggesting that cells within the explant could be spreading or proliferating (n = 14, Fig. 5B, control). In contrast, posterior streak explants contained motile cells, which actively migrated away from the explants (n = 13, Fig. 5C, control). Primitive streak explants dissected from later stages (HH10-12) remained cohesive and contained few individually migrating cells (Fig 5D).

### Wnt3a and Wnt5a affect the behaviour of cells in primitive streak explants

Next we examined whether Wnt3a and Wnt5a could affect cell behaviour by exposing GFP labelled primitive streak explants to pellets of Wnt expressing cells. Time-lapse movies showed that Wnt3a had no discernable effect on anterior primitive streak explants. The explants increased in size but no individual cells moving away from explants were observed (n = 8, Fig. 5B, D). In contrast, the behaviour of cells was altered in posterior streak explants in response to Wnt3a. The majority of explants remained compact with only few individually migrating cells (n = 11, Fig. 5C, D; p < 0.0001). This suggested that Wnt3a inhibited individual cell movements.

When we examined the effects of Wnt5a expressing cells on the behaviour of explants, we observed reciprocal phenotypes. Wnt5a did not alter the behaviour of posterior explants from their normal behaviour and in the majority of cases we observed cells that migrated away from the graft (n = 13, Fig. 5C, D). However, when anterior explants were exposed to Wnt5a their behaviour was changed and individually migrating cells were observed in a significant number of explants (n = 17, Fig. 5B, D; p = 0.0003).

As Wnt3a and Wnt5a appeared to have opposing effects on cell migration we next exposed explants from either anterior or posterior primitive streak to both Wnt3a and Wnt5a expressing cells. In the majority of cases no migrating cells were observed (n = 18 anterior, n = 15 posterior) (Fig. 5B, C, D) suggesting that the ability of Wnt3a to suppress migration was dominant over the ability of Wnt5a to induce migration in these explants. These results were statistically significant as shown by χ^2 ^analyses and a quantitative representation is shown (Fig. 5D).

### Different Wnt pathways affect movement behaviour of primitive streak cells

**Figure 6:**
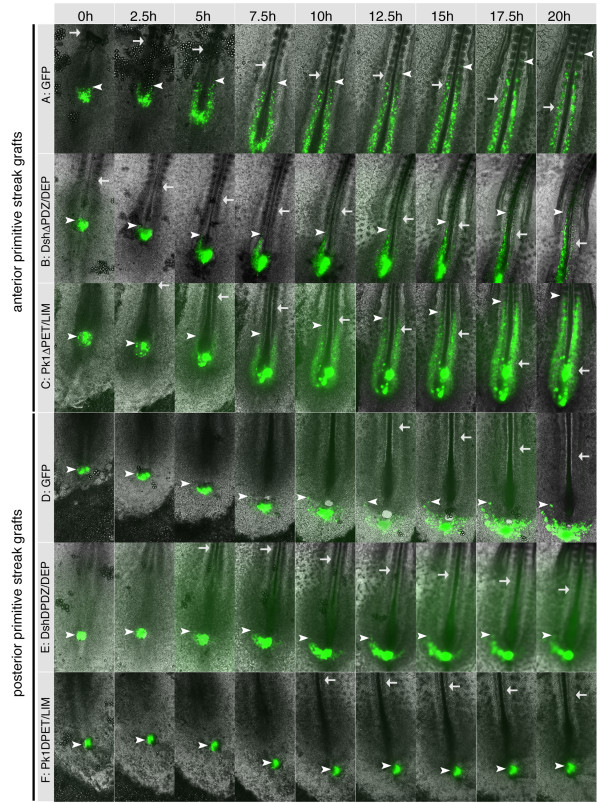
**Wnt signalling components differentially required for generation of mesoderm from the late primitive streak**. Timelapse imaging of embryos electroporated and grafted into unlabelled host embryos with GFP over 20 hours – intervals of 2.5 hours are shown. GFP control cells grafted into the anterior primitive streak(A) or into the posterior primitive streak (D). Cells expressing pCAβ-IRES-GFP-DshΔPDZ/DEP grafted into the anterior streak (B) or into the posterior streak (E). Cells expressing pCAβ-IRES-GFP-Pk1ΔPET/LIM grafted into the anterior streak (C) or into the posterior streak (F). Arrows show the position of the most recently formed somite. Arrowheads indicate the anterior-most GFP positive cells.

We generated constructs to disrupt downstream effectors of Wnt signalling to investigate which pathways control the movement behaviour of primitive streak cells. These were cloned into the pCAβ-IRES-GFP plasmid, which expresses green fluorescent protein from the same backbone [26]. This allowed tracking of transfected cells. Embryos were electroporated between HH8 and 10 and GFP positive cells were grafted homotypically into the anterior or posterior streak of stage-matched host embryos. Time-lapse movies of control grafts labelled with pCS2^+^GFP showed movement behaviour indistinguishable from that seen using DiI and DiO labelling (Fig. 6A, D). At the end of imaging, cells that had originated from the anterior streak were typically seen within paraxial mesoderm and in up to 6 somites (Fig. 6A), compared to DiI labelled cells, which typically contributed to the last 6–8 somites formed (Fig. 3A). Similarly, GFP positive lateral plate mesoderm cells from the posterior streak had clearly undergone lateral movement (Fig. 6D), but had not migrated as far into the lateral plate as DiO labelled cells (Fig. 3A). These differences probably reflect a lag period following electroporation and grafting of GFP expressing cells into a host embryo compared to dye injections.

We wished to examine the role of downstream components of the Wnt signalling pathway and so we employed a dominant negative dishevelled construct (Dsh1ΔPDZ/DEP-IRES-GFP) [27] to establish its function in the late streak. Electroporation of Dsh1ΔPDZ/DEP-IRES-GFP did not affect the emergence of nascent mesoderm and cells were observed leaving the primitive streak (Fig. 6B, E). However, subsequent axis extension was impaired, particularly in anterior primitive streak grafts (Fig 6B). In these embryos, Dsh1ΔPDZ/DEP-IRES-GFP expressing cells did not move normally, their contribution to paraxial mesoderm was impaired and at the end of imaging they were only found in the last 3–4 somites (Fig. 6A, B). Electroporation and grafting of Dsh1ΔPDZ/DEP-IRES-GFP expressing cells into the posterior streak resulted in normal lateral migration of cells during the imaging period (Fig. 6E).

**Figure 7:**
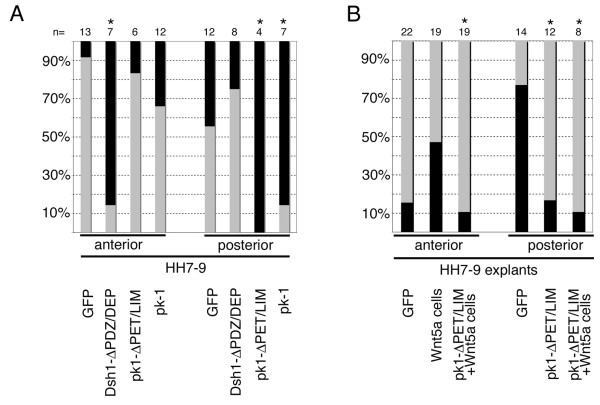
**Electroporation of pk1ΔPET/LIM affects the migration of primitive streak cells in vivo and in explants**. (A) Quantitative representation of in vivo grafting experiments shown in Fig. 6. The normal behaviour of anterior and posterior explants, expressing GFP is represented as light grey shading. Dsh1-**Δ**PDZ/DEP disrupts normal behaviour anteriorly but not posteriorly. pk1-**Δ**PET/LIM and pk1 constructs disrupt normal behaviour posteriorly but not anteriorly. (B) Streak explants containing migratory cells are represented with black shading. Exposure of anterior explants to Wnt5a can induce migratory behaviour but this is abolished in explants expressing pk1**Δ**PET/LIM. The majority of posterior explants expressing pk1-**Δ**PET/LIM are no longer migratory and this cannot be rescued by exposure to Wnt-5a expressing cells. * asterisk indicates statistically significant results.

Next, we investigated the role of pk1 in this context (Fig. 2). Plasmids encoding full-length pk1-IRES-GFP or pk1ΔPET/LIM-IRES-GFP lacking the conserved PET and LIM domains were electroporated. GFP positive cells were grafted into anterior or posterior primitive streak of HH 8–10 embryos and observed by time-lapse imaging. Anterior primitive streak cells mis-expressing pk1 contributed normally to paraxial mesoderm in the majority of cases (Pk1-IRES-GFP, n = 12; Pk1ΔPET/LIM-IRES-GFP, n = 6; Figs. 6C, 7A). Some embryos displayed extension defects similar to those seen with Dsh1ΔPDZ/DEP-IRES-GFP (Pk1-IRES-GFP, 4/12; Pk1ΔPET/LIM-IRES-GFP, 1/6). In contrast, in the posterior primitive streak full-length pk1 (n = 7) or the deletion mutant (n = 4) interfered with the ability of streak cells to migrate away from the streak (Fig. 6F, Fig. 7A). Thus, Pk1-IRES-GFP and Pk1ΔPET/LIM-IRES-GFP altered posterior streak cell behaviour quite differently to Dsh1ΔPDZ/DEP.

Because pk1 expression specifically affected posterior streak cell migration we tested if it could also affect migration behaviour of primitive streak explants (Fig. 7B). Anterior streak explants do not normally contain migrating cells unless they are exposed to Rat-B1a-Wnt-5a cell pellets. However, when anterior explants expressing pk1ΔPET/LIM were exposed to Wnt5a this migratory behaviour was not seen, suggesting that pk1ΔPET/LIM could block this activity of Wnt5a. Compared to controls, posterior explants expressing pk1ΔPET/LIM did not contain migrating cells in most cases (n = 12). The migratory behaviour of posterior explants could not be restored by exposure to pellets of Rat-B1a-Wnt5a fibroblasts in the presence of pk1ΔPET/LIM, suggesting that pk1 function is required for this activity (Fig 7B).

## Discussion

In amniote embryos, elongation of the body axis is driven through the generation and migration of mesoderm from the late primitive streak and its subsequent convergent extension movements towards the midline. Here we present time-lapse analysis of mesoderm cell movements both *in vivo *and in explants and uncover a role for Wnt3a and Wnt5a in controlling movement behaviour of cells from anterior and posterior regions of the late primitive streak. Gastrulation at these later neurula stage embryos (> HH7 in chicken) uses distinct pathways and mechanisms from those employed in younger embryos. In addition, the differential effects of Wnt3a, Wnt5a, Dsh1ΔPDZ/DEP, Pk1 and Pk1ΔPET/LIM illustrate molecular differences in the control of cell movement behaviour of mesoderm progenitors from anterior and posterior regions.

### Movement patterns and behaviour of mesoderm progenitors correlate with their anterior-posterior origin from the late primitive streak

Time-lapse movies show that movement trajectories and fates of mesoderm cells from the late primitive streak correlate with their anterior-posterior position. More anterior cells produce more medial tissues and more posterior cells produce progressively more lateral tissues. This fate map is consistent with previous studies in the chick which made similar findings in younger, gastrula stage embryos [2,3,6,28,29]. Extensive data from non-amniote embryos documented that cell fates and movement behaviour of mesodermal cells vary according to the position along the blastopore. In addition, movement behaviour changed during the course of gastrulation (for review see, [30,31]). This has not yet been directly observed in amniote embryos. The time-lapse analyses provided here demonstrate that movement behaviour varied over time and along the primitve streak. At HH4, anterior streak cells displayed extensive migration [6], however, at HH7-9, cells emerging from anterior regions of the primitive streak seem to be displaced from the streak and do not show extensive lateral migration (Figs. 1B, 3A and 6A). This may indicate that in later stage embryos anterior streak cells move in a more passive fashion and it is conceivable that cell polarization and spreading combined with displacement by the regressing node, rather than individual cell migration, governs their movement. Passive cell displacement has recently been measured in early chick embryos using particle image velocimetry. This work showed that at HH5 there is an increasing cranial to caudal motility gradient, with caudal cells actively migrating away from the primitive streak faster than cranial cells [23]. This is in agreement with our data at HH7-9, where cells from the posterior streak were actively migrating both *in vivo *and in area opaca explants while anterior cells were much less motile (Figs. 1B, C; 3A and 5B, C).

Cell movement behaviour was retained in primitive streak explants cultured on the area opaca. Anterior explants usually contained few or no migrating cells and the graft remained compact, however, it frequently increased in size. This may indicate that the cells within these explants are spreading or alternatively proliferating.

### Wnt3a and Wnt5a coordinate cell movement behaviour in the late primitive streak

Heterotypic grafts showed that primitive streak cells are responsive to cues from their environment. The differential expression of Wnt3a and Wnt5a along the late primitive streak (Fig. 2) and the known phenotypes of knock-out mice [13] prompted us to examine their effects on cell movement behaviour. We found that Wnt3a changed the movement behaviour of posterior cells and inhibited their lateral migration from the primitive streak (Fig. 3C). This most likely reflects its function in anterior cells, which are exposed to high levels of Wnt3a expressed in the streak and the overlying neural plate. Movement behaviour was altered in the absence of detectable changes in cell fate, using three different markers. This suggests that movement defects observed in posterior streak cells are unlikely to be a secondary consequence due to changes in cell fate specification (Fig. 4). However, we cannot exclude the possibility that other molecular markers were affected. Wnt5a transcripts were detected throughout the mesoderm and neuro-ectoderm (Fig. 2D-F) and this may explain why placing pellets of Wnt5a expressing cells into the primitive streak did not affect cell movements *in vivo*.

The behaviour of primitive streak cells was also affected by Wnt3a and Wnt5a expressing cells in explants cultured on the area opaca. Wnt3a cells inhibited cell migration in posterior streak explants, which adopted an anterior, cohesive phenotype, while Wnt5a promoted migration of individual cells from anterior explants (Fig. 5B, C, D). The behaviour of explants cultured on the area opaca was highly reproducible and correlated closely with their antero-posterior origin from the streak. Furthermore, it was consistent with the behaviour of cells in whole embryos. Thus, while we cannot exclude the possibility that there is some effect on cell behaviour due to the culture system, we suggest that our observations using this ex-vivo system are of relevance *in vivo*. We propose that high levels of Wnt3a in anterior streak are likely to be involved in regulating movement behaviour of prospective paraxial mesoderm cells from the late primitive streak, possibly by antagonizing Wnt5a, which we suggest regulates cell movement behaviour of prospective intermediate and lateral plate mesoderm cells. This hypothesis is borne out by the behaviour of explants exposed to both Wnt3a and Wnt5a. Both anterior and posterior explants cultured in the presence of Wnt3a and Wnt5a are non–migratory, suggesting that Wnt3a can inhibit Wnt5a-induced cell migration. This antagonistic activity is not observed at earlier stages of gastrulation (HH < 7, Fig. 3D) consistent with the idea that there is a change in the response of primitive streak cells to Wnt3a at neurula stages. This is in keeping with observations in mice where loss of Wnt3a has no effect on early gastrulation movements (where it is thought to be redundant with Wnt8c) but later production of mesoderm is affected [32]. We propose that Wnt3a has distinct functions in the early primitive streak where, together with Wnt8c, it is required for canonical Wnt signalling during mesoderm production. At later stages (from HH7 onwards) however, it acts to antagonise Wnt5a to control generation of non-migratory paraxial mesoderm precursors in the anterior primitive streak. The molecular mechanisms that govern the switch in response to Wnt3a remain unclear, but they may involve changes in the availability of receptors, co-receptors and intracellular effectors.

### Wnt signalling during extension of the body axis

Both canonical and non-canonical Wnt signalling is known to be required for normal gastrulation movements during development. In particular studies in zebrafish have shown that Wnt5a and Wnt11 act through the PCP pathway to drive convergent extension movements while it was shown in mouse that canonical Wnt signals are required to enable mesoderm cells to exit from the primitive streak [12,33-35]. To analyse the role of Wnt signalling components in late primitive streak stage chick embryos we mis-expressed Dsh1ΔPDZ/DEP. Based on existing mutation analyses [27,36] this construct is likely to disrupt dishevelled functions, although in the absence of biochemical experiments its exact mechanism of action remains to be confirmed. Expression of this construct in the anterior streak did not affect the movement of mesoderm cells out of the streak into the paraxial mesoderm but there were effects on the subsequent elongation of this tissue as the embryos underwent axis extension. This shows that Dsh is required for convergent extension movements in the mesoderm. In the posterior streak we saw no effects using this construct. Although the lateral mesoderm cells produced in the posterior streak do undergo CE during axis elongation this does not occur until later as the cells have to migrate to their lateral position first. This may explain why we did not see effects in posterior cells with the Dsh1ΔPDZ/DEP construct over the timescale observed.

Expression of either full-length or a mutant form of pk1 had very different effects to the Dsh1ΔPDZ/DEP mutant. In posterior cells expression of Pk1 or dominant negative Pk1ΔPET/LIM inhibited cell migration while no effect was seen in anterior primitive streak. Expression of pk1ΔPET/LIM in anterior explants also abolished the ability of Wnt5a to induce migration in these cells while posterior streak explants lost their migratory behaviour when expressing pk1ΔPET/LIM (Fig 7). This suggests a role for Pk1 in mediating Wnt5a activity. Both, loss of function and gain of function prickle constructs showed the same phenotype *in vivo*. This is not unprecedented and has also been observed in zebrafish and *Xenopus*, where full length pk1 and pk1 deletion constructs cause convergent extension defects, presumably due to disruption of cell polarity [16,37].

## Conclusion

Our results are consistent with Wnt signalling acting through multiple distinct pathways during the generation of mesoderm from the late primitive streak in the chick. We propose a working model where Wnt3a in the anterior primitive streak mediates the production of paraxial mesoderm by antagonising Wnt5a thereby controlling the non-migratory behavior of these cells. This is in addition to the known functions of Wnt3a in the primitive streak where, along with Wnt8c, it is thought to provide canonical signals for mesoderm production and segmentation [38,39]. Subsequently a dishevelled-dependent mechanism drives convergent extension movements in paraxial mesoderm prior to the production of somites. Finally, a separate pathway, involving Pk1, is needed for cells in the posterior streak to migrate laterally to generate intermediate and lateral plate mesoderm. This posterior pathway could be driven by signalling from Wnt5a. This hypothesis is consistent with our data and explains the phenotypes of Wnt3a and Wnt5a null mice, where later mesoderm production is disrupted.

## Methods

### Embryo culture, DiI/DiO labelling, electroporation and grafting of pellets

Embryos between HH7 and HH10 were cultured on a filter ring on a semi-solid agarose albumin mixture, preparation of EC cultures was essentially as described in [5]. Briefly, embryos and associated membranes were lifted off the yolk using a square piece of 3MM Whatman filter paper with a clover leave shaped hole in the centre. Embryos were rinsed in PBS to remove any yolk and placed on top of an albumin/agarose mixture in a 3 cm Petri dish.

Injection of dyes was performed in EC culture with the embryo facing dorsal side up and the viteline membrane removed. DiI (Molecular Probes, C-7001) 1 mg/ml in ethanol was mixed with an equal volume of 0.3 M sucrose in PBS and warmed to 37°C before injection. DiO (Molecular Probes, D275) was injected at 2.5 mg/ml in DMF and warmed to 37°C before injection. Dyes were loaded into a glass capillary needle and injected into the primitive streak using an Eppendorf Femtojet microinjector. Embryos were then washed in simple saline before imaging.

Electroporation was performed in EC culture with the embryo facing ventral side up. DNA at 1mg/ml was mixed with fast green to allow visualization of the injection and injected into the primitive streak using an Eppendorf Femtojet microinjector. Electrodes were made from 1 mm diameter platinum wires bent into an L-shape. These were placed above the area to be electroporated and ten 50 milli seconds pulses of 15 V were delivered using an Intracel TSS20 Ovodyne elcectroporator. The polarity of the current was reversed and the series of pulses was repeated. Embryos were then incubated at 28°C for 16–20 hours which allowed GFP expression to commence but arrested embryo development. Grafts of anterior and posterior primitive streak were dissected using tungsten needles and transferred into unlabelled hosts.

Rat-B1a fibroblasts expressing Wnt or control cells containing the empty LNCX vector were previously used in [25]. They were grown in selective medium containing 250 mg/ml G418, and pellets were generated by plating cells on bacterial Petri dishes over night. Pellets were transferred using a Gilson Pipette and pushed into the primitive streak into a small slit created with a tungsten needle. When cell pellets were grafted to one side of the streak, we found that during axis extension the site of labelling in the primitive streak moved away from the grafted pellet during morphogenetic movements. Therefore to keep the labelled cells in close proximity to the grafted cell pellet it was necessary to graft into the streak itself.

### Primitive streak cell explants

Small grafts were dissected from anterior or posterior primitive streak of HH7-9 embryos unless stated otherwise in the text, transferred to a HH4 host embryo using a Gilson Pipette, placed on the area opaca and pushed into a small slit created with a tungsten needle. Wnt expressing cells were grafted adjacent to the explants. Explants were scored for migratory or non-migratory behaviour based on the presence or absence of individual GFP labelled cells migrating out from the explant. In the absence of any migratory cells the explants were scored as non-migratory.

### Long-term video microscopy

Embryos in EC culture and explants on the area opaca of HH4 embyros in EC culture were imaged on a Zeiss Axiovert using a Zeiss Axiocam HRm and Axiovision software. GFP and DiO were visualised with Zeiss filter set 13 (excitation 460–480 nm, emission 505–530 nm) and DiI was visualised with Zeiss filter set 00 (excitation 530–580 nm, emission 615 nm LP). Images were captured every 6 minutes for a period of 20 hours. Files were exported as pseudo-coloured AVI movie files using Zeiss Axiovision software and converted to Quicktime movie files using Quicktime with the H263 compression algorithm.

### Cloning of expression constructs

Full-length cDNAs were generated for c-Dsh1, c-Pk1 by RT-PCR [40] using whole embryo cDNA from 3- and 4-day embryos as template. All primers used contained restriction sites for cloning into pCAβ-IRES-GFP [26] and reverse primers included sequences to encode an HA-tag to confirm protein expression following transfection of HEK293 cells by western blot (not shown). The primers were: chick-Dsh1F: GGATCCATGGCGGAGACTAAAATCATC, chick-Dsh1R: GCGGCCGCTCAAGCGTAATCTGGAACATCGTATGGGTACATGATGT CGACAAAGAATTC, chick -Pk1F: GGATCCATGGAGCCCAAAGCTAAC, chick -PK1R: GCGGCCGCTCAAGCGTAATCTGGAACATCGTATGGGTAAGAAATTATGCAATTTTC PCR products were ligated into pGEMT-Easy (Promega) and sequenced. Chick Pk1ΔPET/LIM was generated by inserting an extra *Stu*I restriction site at position 915–920. Digestion with *Stu*I removed sequences encoding the PET and LIM domains (amino acids 36 – 305) and the remaining plasmid was religated to produce the deletion mutant Pk1ΔPET/LIM. Chick Dsh1ΔPDZ/DEP was generated by using an alternative reverse primer, which introduced a stop codon after amino acid 143: chick-Dsh1ΔPDZ/DEP-R: GCGGCCGCTCAAGCGTAATCTGGAACATCGTATGGGTAG GACATGTGGAATCAGTG.

### In situ hybridisation

*In situ *hybridisation was performed as described [41]. Full length chick cDNAs for Wnt3a, Wnt5a and Wnt8c were generated by PCR and cloned into pGEMT-Easy (Promega) using the following primers: cWnt-3aF+Xbal: TCTAGAATGAAGTCGT TCTGCAGCGAAG, cWnt-3aR+SmaI:

CCCGGGTCAAGCGTAATCTGGAACATCGTATGGGTATTTGCACGTGTGGACGTCGTAG, cWnt-5aF+Xbal: TCTAGAATGGAGAAATCCACTGCAG, cWnt-5aR+NotI:

GCGGCCGCTCAAGCGTAATCTGGAACATCGTATGGGTATTTGCACACAAACTGGTCCAC, cWnt-8CF+Xbal: TCTAGAATGAGGGGCAGCACCTTCCTC, cWnt-8CR+NotI:

GCGGCCGCTCAAGCGTAATCTGGAACATCGTATGGGTATCTCCTGTGGCCTTTGTTCCG. Probes for Wnt3a, Wnt5a, Wnt8c, pk-1, dishevelled-1, dishevelled-3, brachyury and Lef-1 were generated by linearizing pGEMT-Easy template and transcribing with the following enzymes; Wnt3a: *Bam*HI, SP6; Wnt5a, *Nco*I, SP6; Wnt8c, *Nco*I, SP6; Pk1, *Sal*I, T7; Dsh1, *Nde*I, T7; Dsh3, *Sal*I, T7, c-Bra, *Xho*I, T3, Lef1, *Bam*HI, T3.

## Authors' contributions

DS performed the time-lapse analyses, *in situ *hybridisation and drafted the manuscript. OC generated the pk1 constructs and LW generated the Dsh1 constructs and provided the pk1 *in situ *hybridisation. CW participated in the design of the study and AM conceived and co-ordinated the study and produced the manuscript.

## Supplementary Material

Additional file 1 Migration of cells from anterior and posterior primitive streak is shown, following labelling with DiI (red) in the anterior streak and DiO (green) in the posterior streak. Anterior cells do not migrate laterally but move into the paraxial mesoderm where they undergo convergent extension. Posterior cells migrate laterally before undergoing convergent extension movements. The image sequence shown in Fig 3A was generated from this movie. Click here for file
